# Patterns of Active Travel and Physical Activity among Adolescents in Israel

**DOI:** 10.3390/ijerph192114115

**Published:** 2022-10-28

**Authors:** Sharon Levi, Orna Baron-Epel, Riki Tesler, Yossi Harel-Fisch

**Affiliations:** 1 School of Public Health, University of Haifa, Haifa 3498838, Israel; 2 Efsharibari-The Israel National Program for Active & Healthy Living, Ministry of Health, Jerusalem 9101002, Israel; 3 The Department of Health Systems Management, Faculty of Health Sciences, Ariel University, Ariel 40700, Israel; 4 International Research Program on Adolescent Well-Being and Health, Faculty of Education, Bar-Ilan University, Ramat Gan 5290002, Israel

**Keywords:** active transport, walking, cycling, e-cycling, family activities

## Abstract

Active travel (AT) is a core physical activity (PA) indicator for children and youth; contributing to health and wellbeing, at both the individual and societal levels. This analysis explores patterns of adolescent active school travel (AST) and cycling and associations with different PA measures. Secondary analysis of the cross-sectional 2018–2019 Health Behaviour in School Age Children study in Israel included an extended PA module with walking, cycling and e-cycling modes. The nationally representative sample includes students in grades 6–12 (*n* = 4407). Analysis of weighted data included descriptive analyses, inferential statistics, and regression analyses. AST was reported by 61.9% of adolescents; 39.3% reported 20 min or more daily AST; 27.1% reported habitual cycling (HC) and 17.4% reported habitual e-cycling (HEC). There are mixed results for socio-economic status and environment. AST and HC were associated with less sedentary hours daily (odds ratio [OR] = 1.190 and 1.397, respectively); HC was associated with positive sports self-rating (OR = 2.394). Stepwise regression analysis found that lower AST duration, time in minutes, was associated with watching television with the family. Promotion of adolescent AT may be associated with increased PA and joint parent-adolescent AT, and was utilized across different socio-demographic groups in Israel.

## 1. Introduction

Physical activity (PA) is a key component to improving health outcomes among adolescents. Regular participation in PA can help reduce the risk of chronic disease including cardiovascular disease, diabetes, certain cancers, and hypertension [1]. PA is also linked to positive mental health among children and adolescents, including outcomes related to depression, anxiety, self-esteem and cognitive functioning [2]. In addition, there is evidence of favorable relationships between PA and several cardiometabolic biomarkers (e.g., cholesterol, blood pressure, triglycerides), adiposity, physical fitness (aerobic fitness, muscular strength, and endurance), and bone health [3]. The reduction of sedentary behavior among adolescents is also related to health benefits [4]. This is due to the fact that higher frequency and duration of different sedentary behaviors, including screen time and gaming, are associated with several negative health indicators, including higher body mass index, cardio-metabolic risk factors, and lower fitness. They are also associated with unfavorable behaviors, poor interpersonal communication, and lower self-esteem [5]. However, in spite of the benefits of PA, international studies show that four in five adolescents are not achieving the minimum recommendations of 60 min of moderate to vigorous PA daily [6].

Active travel (AT) such as walking or cycling, is one method to increase overall PA and is considered a core PA indicator for children and youth, as well as for the general population [7,8]. Efforts to improve conditions and rates of AT have become a policy priority in many countries due to the potential to improve health outcomes and wellbeing at an individual level, as well as providing societal and environmental benefits in a community as a whole [8,9,10]. Studies in different countries have found that higher levels of active travel are associated with lower levels of BMI or obesity rates [11,12,13]. Research has shown that AT is related to higher rates of PA among children and adolescents [14,15,16]. There are also indications that AT is associated with improved physical fitness [17]. However, many countries show a significant decline in AT, including child and adolescent travel over the past decades [18]. Moreover, in a comparison of child and youth PA measures across 30 countries with a very high human development index, the majority report 50% or less of AT among children and adolescents nationally [19].

Previous research indicates that higher rates of AT among adolescents are associated with a variety of environmental, psychosocial and safety factors. In a systematic review of research on AT to school, AT was found to be associated positively with safety, walkability and neighborhood social interactions, and negatively with travel distance and car ownership [18]. Similarly, a review of the association between the built environment and both child and adult AT found that walkability, parks and playgrounds, improved infrastructure for AT, and safe places to walk had a positive effect on AT [20]. In a study of transportation mode choice by adolescents in Cyprus, travel time and cost were found to affect mode choice behavior and AT was preferred when facilities such as bicycle paths, stands and wide sidewalks are available [21]. Key psychosocial factors that have been cited as being related to adolescent AT including self-efficacy, social support from peers and parents, and enjoyment of PA [22]. Perceptions of safety, both of parents and adolescents, including dangers of traffic and crime have been found to be negatively associated with independent AT [23,24]. Demographic correlates with active school travel (AST) include negative association with increase in age, female gender, household income and car ownership [18,25]. Evidence of the association of child and adolescent AT and ethnicity in North America found that AST was positively associated with respondents who self-identified as Hispanic [25].

A unique mode of AT that has increased among adolescents in Israel is the electric bicycle (e-bike): bicycles with pedal-assisted electrical support that can reach speeds of up to 25 km per hour. The Israel Tax Authority reported that, between 2014–2018, over 320,000 e-bikes were imported to Israel, reaching approximately 16% of bicycle imports (there is no local bicycle production) [26]. A study of e-bike use by adolescents found that this mode may meet a need for mobility independence among adolescents and serve as an alternative to personal vehicles [27]. This is in line with research that e-bikes may promote adoption of active travel reducing common barriers to other active travel modes, including distance and fatigue [28,29]. A systematic review of the health benefits of e-bikes found that there is moderate evidence that e-bikes contribute to moderate intensity PA for adult riders [29]. Hospital and trauma studies point to an increase in injuries related to e-bike use, in particular for young riders, this increase is attributed to increased use of e-bikes as well as potential risky behaviors [26,30]. In a study of naturalistic behaviors for a large population of children and adolescents at urban intersections in three cities, e-bikes were found to be associated with higher rates of risky behaviors including crossing without checking traffic at non-signalized junctions and crossing on red lights at signalized intersections [31]. Similarly, in another study of e-cycling behaviors among adolescents in Israel, observed behaviors included non-use of helmets in contradiction with national regulations, crossing on red, higher speeds and other risky maneuvers in the urban environment [32,33]. Research on adolescent AT in Israel is limited and has primarily focused on specific urban areas. For instance, a study with 5th and 6th graders in a large municipality, found that higher rates of walking were identified in the more highly urbanized areas; however, cycling was negatively related to measures of urbanicity such as intersections and residential density [34]. Previous studies of adolescent and child AT have not included different child and adolescent racial/ethnic populations in Israel. Each population group has different socio-cultural identities, practices, and resides in different types of settings, all of which may affect different health behaviors. Accordingly, previous research pointed to a higher rate of PA among Jewish students in Israel [35].

The current study was undertaken to determine prevalence of adolescent AT, based on measures of AST, habitual cycling (HC), and habitual e-cycling (HEC), using a nationally representative sample. The analysis serves to identify different characteristics related to adolescent AT, as well as the association between AT and different PA measures. The findings from the current analysis will contribute to an understanding of the use of AT by adolescents in Israel and the role of AT in relation with other PA behaviors.

## 2. Materials and Methods

### 2.1. Data Source and Participants

Analyses utilized the most recent cycle of the Health Behaviour in School-Aged Children (HBSC) Study conducted during the 2018–2019 school year [36]. HBSC is a World Health Organization (WHO) affiliated, cross-national study with 11–15-year-olds in 50 countries. It studies health behaviors, well-being, and their social context and determinants. It employs an international mandatory questionnaire with additional modules that are used by sub-samples of participating countries. The most recent cycle in Israel included both a module on AST as well as an in-depth and extended PA module [37].

In order to ensure a representative sample, and in accordance with the international HBSC protocol, 227 schools across Israel, representing different subpopulations and geographic regions, were randomly selected from the Israel Ministry of Education list of schools [37]. Classrooms were randomly sampled (90% classroom response), and for each sampled school, an additional class was also randomly sampled. All students who were present in sampled classrooms were included (>95% pupil response). Sample weights were calculated to ensure relatively accurate representations of the subpopulations in Israel in the sample. The total sample for the 2018–2019 study was 13,845 students 11 to 17 years old; the PA module was conducted with a nationally representative subset of 4407 students. The research protocol was approved by the ethics committees of the Israeli Ministry of Education and Bar Ilan University (No. 10203).

### 2.2. Key Measures

#### 2.2.1. Dependent Variables

Active travel is the key exposure of interest and is based on measures of AST, habitual cycling (HC), and habitual e-cycling (HEC).

**Active School Travel**

The measure is based on the question ‘On a typical day is the main part of your journey to school made by…?’ and ‘On a typical day is the main part of your journey from school made by…?’ with the possible answers (1) walking; (2) bicycle; (3) e-bike; (4) public transport; (5) car, motorcycle, or moped; (6) other means. The transport option e-bicycle was added in the past two waves of the HBSC study, as this has been adopted as a common mode of travel for adolescents in Israel [27]. We created a dichotomous variable called AST: respondents who indicated “walking”, “cycling” or “e-cycling” as their main method of transport to or from school were classified as engaged in AST (1); while respondents who did not report usually using one of these three modes either to or from school were categorized as not using AST (0). In previous research, AST was tested and found to have a high reported level of agreement between participants (Cronbach’s alpha ≥ 0.80) [38].

A measure of AST duration was classified based on the question “How long does it usually take you to travel to school from your home?” Response options were (1) less than 5 min, (2) 5–15 min, (3) 15–30 min, (4) 30 min to 1 h and (5) more than 1 h. The AST duration was calculated based on the average daily minutes of AST, including walking, cycling and e-cycling, based on trips to and from school. A similar measure of AST duration was tested psychometrically and found to have a high percentage of agreement between participants (range 74–96%) [39].

**Habitual Cycling**

We examined the amount of time spent cycling on a regular basis, not just for AST. The variable was based on the question, ‘In the past year how often did you ride a bicycle (regular, not e-bicycle)?’ The possible answers included (1) almost every day; (2) once or twice a week; (3) seldom; (4) never. We created a dichotomous variable called HC; respondents who indicated response options ‘almost every day’ and ‘once or twice a week’ were classified as engaged in HC (1); while respondents reported that they ‘seldom’ or ‘never’ ride a bicycle were categorized as not engaged in HC (0).

**Habitual E-cycling**

We examined the amount of time spent e-cycling on a regular basis, not just for AST. The variable was based on the question, ‘In the past year how often did you ride an e-bike?’ The possible answers included (1) almost every day; (2) once or twice a week; (3) seldom; (4) never. We created a dichotomous variable called HEC; respondents who indicated response options ‘almost every day’ and ‘once or twice a week’ were classified as engaged in HEC (1); while respondents reported that they ‘seldom’ or ‘never’ ride an e-bike were categorized as not engaged in HEC (0).

#### 2.2.2. Independent Variables

The primary outcome measure of interest was PA based on several indicators. 

**Physical activity**

The primary PA indicator was measured based on the number of days reported with 60 min of overall PA, based on the WHO recommendation of 60 min a day of moderate-vigorous intensity PA (MVPA) for children and youth. Respondents indicated the number of days in the previous seven days in which they engaged in at least 60 min/day of MVPA. We grouped the variable into three levels: none (0 days); some (1–5 days); and frequent (6–7 days). Six and seven days were grouped together due to the unique religious characteristics across different population groups in Israel (Jewish and Arab) that may prevent engagement in PA on a seventh day of the week. This question has been validated against accelerometer data and been tested for reliability and validity in multiple studies. It has been found to be acceptable for measuring adolescent PA recommendations [40].

**Self-rating of sports skills**

Respondents indicated whether they considered themselves to be good at sports in comparison with their peers. The self-rating measure allowed for the following response options: (1) one of the best, (2) good, (3) average, and (4) less than average. 

**Sedentary behavior**

Respondents indicated how many hours on average of free time (i.e., outside of school) on weekdays were spent sitting. Examples provided included watching television, using the computer, or riding in a vehicle. Response options ranged from zero to seven hours daily. We created a dichotomous variable, based on findings for the mean sedentary behavior among respondents. Youth reporting sitting for four or more hours on average were classified as ‘Sedentary 4+ hours daily’ (1); youth reporting sitting for less than four hours on average were classified as ‘Sedentary 4< hours daily’ (0). 

**Joint Family Activities**

An additional independent variable of interest was the relationship between AT and a variety of family activities. Youth were asked about a series of activities and the rate at which they engage in these activities with their family. The eight activities include: watching television or movies, playing games at home, eating meals together, going for walks together, going places together, visiting friends or family, engaging in sports together, and sitting together to chat. The possible options include: (4) every day; (3) most days of the week; (2) once a week; (1) less than once a week; and (0) never. For the purposes of descriptive analysis and tests for statistical significance the measures were converted to dichotomous indicators to reflect joint activities every day or most days of the week (1); once a week or less (0).

#### 2.2.3. Covariates

Possible confounders of the relationship between AT and PA have been documented in previous literature on these health behaviors [18,41,42]. Confounders available for study included gender, grade, and adiposity (based on body mass index and categorized using age- and gender-specific cut-points for obese respondents). Socioeconomic status was based on the family affluence scale, a validated HBSC measure based on a composite sum score derived from participant responses to four items: number of household bedrooms, number of annual family holidays, family vehicle ownership, and computer ownership [40]. Vehicle ownership was also analyzed separately. School affiliation was included, as it represents three distinct populations in Israel: Jewish-Secular, Jewish-Orthodox, and Arab. As a measure of the environment, respondents were presented with a list of nine different categories of AT and PA facilities, and they were asked whether each of these facilities were available near their home or in their neighborhood. The facilities include: walking or cycling paths, a playground or park for playing, a public basketball court or soccer field, a school gym available for use outside of school hours, a skate park, public exercise equipment, a public pool, a community center with sports classes, or a tennis court (Cronbach’s Alpha 0.812). A score was created based on three levels of facility availability: (1) low (0–2 facilities); (2) medium (3–6 facilities); and (3) high (7–9 facilities).

### 2.3. Statistical Analysis

Descriptive analyses were performed as the first step. They were followed by tests for statistical significance of observed group differences using analysis of variance (ANOVA). We then used multiple logistic regression to explore the associations between AST and HC and key measures of PA. 

To explore the association between AST duration and activities with the family, a backwards stepwise approach was used to test interactions, where all eight terms were entered into an initial model and terms with the largest *p* value were removed one at a time until only terms with *p* < 0.05 remained. In order to ensure that the AST duration variable will be less skewed and to allow for ordinary regression, we calculated the square root of the variable and transformed to z-scores. A *p* value of 0.1 was used to interpret significance of interactions. 

Prior to the analysis a variance inflation factor (VIF) test was used to check the terms for each of the models for multicollinearity; as no VIF score exceeded 2.2 and the tolerance was not found to be below 0.46 for any of the variables we concluded that there was no threat of multicollinearity.

We performed analyses using SPSS version 27.0 (IBM SPSS Statistics, Armonk, NY, USA). Statistical significance was set at 0.05 unless otherwise indicated.

## 3. Results

### 3.1. Participant Characteristics

The total sample available for analysis was 4407 students. Approximately equal numbers of male and female students participated, and there was a similar representation across the different grades representing young and older adolescents, with grades 11–12 grouped together in the study (Table 1). 

Regular use of AST, including walking, cycling and e-cycling, was reported by 61.9% of adolescents, walking being the most common mode of AST, followed by e-cycling and cycling. Among students engaged in AST, 39.3% reported 20 min or more of daily AST. In addition, 27.1% of students reported HC and 17.4% reported HEC. 

A total of 14.3% of the adolescents reported a frequent rate of 60 min of PA a day, while 21.6% reported that they did not participate in 60 min of PA any days of the week. Moreover, 37.2% of adolescents reported four or more hours of daily sedentary activity.

The most common joint family pastimes reported by adolescents included sedentary activities such as eating meals together, sitting together to chat, and watching television (73.2%, 64.2%, and 59.5%, respectively). In contrast, engaging in sports and going for walks were the least common reported joint family activities (22.3% and 22.2% respectively).

### 3.2. Associations between Active Travel, Physical Activity and Key Characteristics

The results of the ANOVA analyses, indicated that AST, HC and HEC were associated with gender (higher for males; F(1, 4250) = 35.09, *p* < 0.001 for AST; F(1, 4405) = 341.12, *p* < 0.001 for HC; F(1, 4405) = 150.85, *p* < 0.001 for HEC); age (for AST and HC highest in grade 6; F(3, 4248) = 15.11, *p* < 0.001; F(3, 4403) = 71.85, *p* < 0.001; for HEC highest in grades 10 and 6; F(3, 4405) = 3.25, *p* < 0.05); school affiliation (highest for Arab schools; F(2, 4249) = 43.77, *p* < 0.001; F(2, 4404) = 32.75, *p* < 0.001; F(2, 4405) = 23.96, *p* < 0.001). None of the measures of AT were significantly associated with socio-economic status, vehicle ownership or adiposity. There was an association between AT and PA facilities in the environment and AST and HEC, but not HC; both AST and HEC were associated with a low reported level of facilities (F(2, 4249) = 20.82, *p* < 0.001; F(2, 4405) = 9.557, *p* < 0.001). Correlations were identified between AST, HC and HEC and different PA indicators: higher rates of frequent PA days a week (F(2, 4249) = 8.87, *p* < 0.001; F(2, 4404) = 69.02, *p* < 0.001; F(2, 4405) = 9.76, *p* < 0.001) and report that they are as good or better than their peers at sports (F(3, 4248) = 5.47, *p* < 0.001; F(3, 4403) = 60.07, *p* < 0.001; F(2, 4405) = 46.05 *p* < 0.001). Conversely, all three measures were negatively associated with sedentary behavior (F(1, 4250) = 23.45, *p* < 0.001 for ATS; F(1, 4405) = 57.98, *p* < 0.001 for HC; F(2, 4405) = 20.10, *p* < 0.001 for HEC). 

Table 2 summarizes the logistic regression analyses that describe the association between engagement in AST and HC and the different demographic and PA measures. Findings for HEC were similar to HC and are not presented in the table.

A statistically significant positive relationship was observed for both AST and HC for males (ORs = 1.463 and 3.381, respectively) and grade (highest for grade 6; ORs = 1.311 and 2.821, respectively). Socio-economic status using the family affluence scale was only found to be associated with HC for the higher group (OR = 1.299). We found a statistically significant positive relationship between school affiliation and both AST (Jewish-Secular and Arab schools; ORs = 1.905 and 2.001, respectively) and HC (Arab schools, OR = 2.252). AST and HC were associated with fewer sedentary hours daily (ORs = 1.190 and 1.397, respectively). A lower rate of days with 60 min PA was associated with a lower rate of both AST (none, OR = 0.723) and HC (none, and some; ORs = 0.508 and 0.675, respectively). HC was also associated with higher self-rating for sports (highest for rating ‘One of the best’ OR = 2.394). A statistically significant positive relationship was observed for AST and a low rating for number of AT and PA facilities in the environment in their town (OR = 1.460). Conversely, HC had a negative association with a low rating for facilities in the environment (OR = 0.694). Most of these results are consistent with the ANOVA analyses.

Regarding joint family activities, there were positive correlations for AST, HC and HEC with most of the reported behaviors. The three measures were associated with frequently playing games at home (F(1, 4250) = 9.00, *p* < 0.01 for AST; F(1, 4405) = 103.67, *p* < 0.001 for HC; F(1, 4405) = 19.86, *p* < 0.001 for HEC), eating meals together (F(1, 4250) = 15.21, *p* < 0.001; F(1, 4405) = 38.63, *p* < 0.001; F(1, 4405) = 5.10, *p* < 0.001), going for walks together (F(1, 4250) = 13.35, *p* < 0.001; F(1, 4405) = 111.82, *p* < 0.001; F(1, 4405) = 81.73, *p* < 0.001), going places together (F(1, 4250) = 11.21, *p* < 0.001; F(1, 4405) = 76.87, *p* < 0.001; F(1, 4405) = 38.63, *p* < 0.001), visiting friends or family (F(1, 4250) = 6.89, *p* < 0.01; F(1, 4405) = 71.10, *p* < 0.001; F(1, 4405) = 42.66, *p* < 0.001), engaging in sports together (F(1, 4250) = 16.39, *p* < 0.001; F(1, 4405) = 123.77, *p* < 0.001; F(1, 4405) = 72.13, *p* < 0.001). AST and HC were associated with and sitting together to chat (F(1, 4250) = 4.70, *p* < 0.05; F(1, 4405) = 20.34, *p* < 0.001). Both HC and HEC were positively associated with watching television or movies every day or most days of the week (F(1, 4405) = 25.51, *p* < 0.001; F(1, 4405) = 5.73, *p* < 0.05).

Table 3 presents the backwards stepwise regression analysis that was used to identify the association between different types of activities with the family and AST behavior. 

In the sixth step the model found that the two remaining activities related to higher AST duration were walking with the family and eating meals together and in the final step eating meals together remains. Lower AST duration was associated with watching television with the family identified in the sixth and seventh step. Analysis conducted prior to calculation of square root of the variable and transformation to z-scores found similarly that in the sixth step of the model the two remaining activities related to higher AST duration were walking with the family and eating meals together and in the final step walking with the family remains. By calculating the square root we reduced the effect of extreme values on the results which may effect the results. Similar findings were found for AST frequency. Among the various joint family activities, watching television or movies is negatively associated with adolescent engagement in AST.

## 4. Discussion

We aimed to document national rates of adolescent AST and cycling and the association of these measures with PA, as well as with a variety of demographic, environmental, and psychosocial characteristics in order to better understand their associated factors among adolescents in Israel. Consistent walking, cycling or e-cycling to and from school, as well as leisure cycling and e-cycling on a regular basis, was reported by adolescents. The findings demonstrate that the prevalence of adolescent AT in Israel is higher than average as reported for countries with very high scores on the Human Development Index [19]. Moreover, many adolescents reported 20 min or more of daily AST representing a significant distance, whereas perceived travel distance and time have been found to negatively affect AST [18]. Use of e-bikes for AST was more common than regular cycling. Transition to e-cycling in late adolescence may afford mobility independence, while maintaining AT behaviors in particular in urban areas [27].

We found that AST, HC and HEC were higher for males and AST and HC were highest for younger students in Israel, similar to previous research in several other countries [43]. Interestingly, HEC was found to be highest for high-school age, in line with regulations that allow for e-bike use from age 16, however HEC was also associated with higher use rates among 6th grade respondents in violation of these requirements. In considering the different population groups in Israel, respondents in Jewish-Secular and Arab schools were more likely to engage in AST; however, this may primarily be due to the geographic dispersion of Jewish-Orthodox schools, which often require use of motorized transport. Higher rates of AST for respondents from Arab schools is in contrast with previous research citing lower reported rates of PA as compared to Jewish adolescents [35]. Findings for socio-economic status and the environment measure, relating to number of AT and PA facilities, were mixed. Socio-economic status was not found to be associated with AST or HEC, however a low rating of number of facilities in the environment was associated with AST and HEC; this finding is in contrast with similar studies [18]. This is in contrast to findings related to participation in organized sports in high income countries which is associated with children and adolescents in higher socio-economic households [44]. However, we found that HC was related to higher socio-economic status and associated with a high rating for number of facilities in the environment. This variability points to the complexity of AT: it is dependent on a variety of factors at the individual and environmental level. While several studies point to a positive association between AST and higher neighborhood socio-economic status, at the individual level, AST has been found to be positively associated with low socio-economic status [18]. The potential for adolescent PA related to AT may be more inclusive across the different socio-demographic groups in Israel as compared to participation in organized sports [35]. The findings may reflect the limitation of the study question that relates to availability of various PA and AT facilities in the neighborhood. Furthermore, the lack of AT facilities in the urban environment across Israel may influence the findings [33]. However, in a previous study of child AT in an urban area in Israel, differences were identified between travel modes: walking was associated with more compact urbanized neighborhoods, while cycling was more common in suburban neighborhoods [34]. 

We found that a lower rate of days per week with at least 60 min of PA was associated with both a lower rate of AST and HC, similar to studies that have found that AT is related to higher rates of PA among children and adolescents [14,15,16]. However, the method of measuring PA requests that respondents add up the total time spent daily on MVPA; therefore, some respondents may include AT within these daily minutes. Consequently, we included analyses of additional related indicators and found that both AST and HC were related with fewer sedentary hours daily, and that HC was associated with higher self-rating for sports. HEC was also associated with positive measures of PA, in view of these findings, efforts to promote AT also via e-cycling while maintaining adolescent safety will be an important consideration for public health. Adoption of e-cycling amongst adolescents in Israel is relatively high and offers a different perspective as compared to adolescent AT in other countries.

This study offered a unique opportunity to study the association between AST and activities with the family. As noted, family activities that incorporated AT and PA were the least commonly reported among Israeli adolescents; families in Israel favor sedentary activities together, such as eating meals, sitting to chat and watching television. We found that there were positive correlations for AST, HC and HEC with most of the reported behaviors; this is in line with previous research that points to the association of joint family activities and healthy behaviors, as well as reduced risk-taking behavior [45,46,47]. The analysis of the association between AST and the various joint family activities points to lower rates of AST duration among families that adopt more sedentary activities; these families may be missing the opportunity to further encourage adolescent AT and PA. These findings contribute to previous research on the role of parental support in increasing adolescent PA. Parental involvement is an important contribution to adolescent health and wellbeing; tailored joint activities may have the potential to increase targeted behaviors [48,49,50]. Conversely, parental objections due to safety concerns and lack of appropriate infrastructure may inhibit support for AT [23]. Previous studies in Israel point to higher rates of traffic injuries for children and adolescents using AT modes in urban areas as compared to adults [30,31]; injury prevention strategies as well as public education for parents and adolescents will be an important consideration for policy makers as part of efforts to promote adolescent AT. 

The strengths of the study include that it was based on a large representative sample of adolescents from different populations in Israel. The HBSC research protocol is based on measures that have been tested previously for validity and reliability. Additionally, various modes and measures of AT were included in the study, representing a broader picture of AT. 

The study also had several limitations. The basis of the analysis was a cross-sectional design which does not allow for determining causality. Further, the data is based on self-reporting, and therefore, may be susceptible to recall bias. In the HBSC study, daily AT is only collected for AST; there is a gap of information related to leisure or after-school trips, in particular for walking. The study does not include factors related to transportation mode choice, such as cost or availability, which would contribute to an understanding of motivations for AT. The AST measure is based on a selection of the main method of transport, which may result in an imprecise measure of daily AT. Similarly, the PA measure asks that respondents add up time spent on MVPA, which may result in misrepresentation of the exact amount of activity and time spent on AST.

## 5. Conclusions

This exploratory research into adolescent AT in Israel using a nationally representative sample suggests that AST, HC and HEC may be associated with increased PA and are utilized across different socio-demographic groups in Israel. Adolescent AT in Israel is comprised of walking, cycling and e-cycling; in a review of AST, HC and HEC we found that these behaviors have the potential to increase overall health and wellbeing at both the individual and societal levels. Inclusion of micromobility modes, such as e-bikes, in measures of active travel is of particular importance in light of the increasing density of the urban centers in Israel and worldwide. Policies in support of appropriate and safe infrastructure and education for AT, such as increased bicycle lanes, safe sidewalks, and training on rules of the road may help to prevent a transition to personal vehicles in late adolescence as well as encourage parental support for AT. Further research on AT in Israel is needed to compare the three modes (walking, cycling, e-cycling) and the variety of trips undertaken by adolescents, to consider the association between AT and additional variables, such as the effects of parents, peers, screen-time habits, and to consider the association of AT with additional measures of physical and recreational activity.

## Figures and Tables

**Table 1:** Demographic characteristics, active travel behaviors, and physical activity indicators; unweighted sample size and weighted percent, Total Sample *n* = 4407.

Characteristic	Unweighted Sample	Weighted Percent
Sex		
Male	1861	50.8%
Female	2546	49.2%
Grade		
6	954	21.2%
8	962	20.6%
10	898	18.8%
11–12	1593	39.5%
Family Affluence Scale		
Low	1012	21.7%
Medium	1722	38.4%
High	1673	40.0%
School Affiliation		
Jewish Orthodox	709	14.2%
Arab	1575	32.0%
Jewish-Secular	2123	53.8%
Environment Score		
Low 0–2 AT and PA Facilities	1828	41.5%
Medium 3–6 AT and PA Facilities	1873	42.5%
High 7–9 AT and PA Facilities	706	16.0%
School Transport Mode ^1^		
Walking	2182	51.7%
Cycling	154	4.3%
E-Cycling	222	5.9%
Public Transport	1147	26.3%
Pupil Transport	559	12.4%
Private vehicle/Motorcycle	937	21.7%
Active School Travel Duration ^2^		
No Active Travel	1763	39.9%
Under 20 Minutes	875	20.8%
20 min or more	1614	39.3%
Cycling		
Daily or 1–2 times a week	1128	27.1%
Rarely or Never	3279	72.9%
E-Cycling		
Daily or 1–2 times a week	725	17.4%
Rarely or Never	3682	82.6%
Physical Activity ^3^		
None (0 days)	953	21.6%
Some (1–5 days)	2824	64.1%
Frequent (6–7 days)	629	14.3%
Self-rating of Sports Skills		
One of the best	1115	25.3%
Good	1528	34.7%
Average	1282	29.1%
Less than average	482	10.9%
Sedentary behavior		
Sedentary 4+ hours daily	1649	37.2%
Sedentary 4< hours daily	2758	62.8%
Joint Family Activities ^4^		
Watch television or movies	2639	59.5%
Play games at home	1630	35.4%
Eat meals together	3281	73.2%
Joint Family Activities ^4^		
Go for walks together	1021	22.2%
Go places together	2157	47.4%
Visit friends or family	1778	38.8%
Engage in sports together	993	22.3%
Sit together to chat	2863	64.2%

^1^ School transport mode for both to and from school—total equals more than 100%. ^2^ Active school travel includes walking, cycling and e-cycling to and from school. ^3^ Physical activity (PA) is based on number of days reported with 60 min of overall PA. ^4^ Joint family activities include frequency of responses of every day or most days of the week.

**Table 2:** Key results for logistic regression models, active school travel and habitual cycling.

Characteristic	Active School Travel		Habitual Cycling	
	Odds Ratio (95% CI ^1^)	*p*-Value	Odds Ratio (95% CI ^1^)	*p*-Value
Gender				
Male	1.463 (1.284–1.668)	0.000 **	3.381 (2.904–3.937)	0.000 **
Female	REF	REF	REF	REF
Grade				
6	1.311 (1.094–1.570)	0.003 **	2.821 (2.303–3.455)	0.000 **
8	0.800 (0.672–0.953)	0.012 *	1.952 (1.582–2.410)	0.000 **
10	1.125 (0.944–1.340)	0.189	1.316 (1.051–1.646)	0.016 *
11–12	REF	REF	REF	REF
Family Affluence Scale				
Low	REF	REF	REF	REF
Medium	0.974 (0.822–1.153)	0.757	1.129 (0.923–1.381)	0.238
High	0.938 (0.784–1.121)	0.481	1.299 (1.050–1.608	0.016 *
School Affiliation				
Jewish Orthodox	REF	REF	REF	REF
Jewish-Secular	1.905 (1.575–2.303)	0.000 **	0.883 (0.706–1.105)	0.277
Arab	2.001 (1.620–2.470)	0.000 **	2.252 (1.767–2.869)	0.000 **
Sedentary Behavior ^2^				
Sedentary 4+ hours	REF	REF	REF	REF
Sedentary 4< hours	1.190 (1.043–1.357)	0.010 *	1.397 (1.187–1.643)	0.000 **
Days of 60 Minutes Physical Activity				
None (0 days)	0.723 (0.571–0.916)	0.007 **	0.508 (0.389–0.662)	0.000 **
Some (1–5 days)	0.958 (0.787–1.168)	0.674	0.675 (0.551–0.827)	0.000 **
Frequent (6–7 days)	REF	REF	REF	REF
Self-rating of Sports Skills				
One of the best	1.109 (0.871–1.412)	0.403	2.394 (1.723–3.326)	0.000 **
Good	1.126 (0.901–1.407)	0.298	2.115 (1.536–2.912)	0.000 **
Average	1.035 (0.828–1.293)	0.762	1.637 (1.177–2.278)	0.003 **
Less than Average	REF	REF	REF	REF
AT and PA Facilities in the Environment				
Low	1.460 (1.182–1.804)	0.000 **	0.694 (0.539–0.893)	0.005 **
Medium	1.074 (0.888–1.298)	0.463	0.954 (0.761–1.196)	0.685
High	REF	REF	REF	REF

ATS Model Summary—Chi-square 206.94; df 16; *p* < 0.001; Nagelkerke R^2^ 0.064; Cox&Snell R^2^ 0.048; HC Model Summary—Chi-square 717.69; df 16; *p* < 0.001; Nagelkerke R^2^ 0.221; Cox&Snell R^2^ 0.150. * *p* < 0.05, ** *p* < 0.01; ^1^ Confidence Interval (CI); ^2^ Sedentary behavior based on daily hours of sedentary activity.

**Table 3:** Backward stepwise regression model activities with family that are associated with duration of active school travel.

Model		β (Standard Error)	95% CI ^1^ for β	Beta	*p*-Value
1	(Constant)	−0.07 (0.06)	−0.18–0.04		0.231
	Watch television or movies	−0.03 (0.02)	−0.06-−0.00	−0.039	0.033
	Play games at home	0.00 (0.01)	−0.03–0.03	0.001	0.955
	Eat meals together	0.06 (0.02)	0.02−0.09	0.058	0.002
	Go for walks together	0.02 (0.02)	−0.01–0.05	0.026	0.245
	Go places together	−0.02 (0.02)	−0.06–0.02	−0.022	0.324
	Visit friends or family	0.00 (0.02)	−0.04–0.04	0.000	0.992
	Do sports together	0.01 (0.02)	−0.02–0.04	0.019	0.377
	Sit together to chat	−0.01 (0.02)	−0.04–0.02	−0.013	0.501
6	(Constant)	−0.09 (0.05)	−0.20–0.02		0.092
	Watch television or movies	−0.04 (0.01)	−0.06–−0.01	−0.043	0.011
	Eat meals together	0.05 (0.02)	0.02–0.08	0.049	0.005
	Go for walks together	0.02 (0.01)	−0.01–0.05	0.027	0.116
7	(Constant)	−0.10 (0.05)	−0.21–0.00		0.053
	Watch television or movies	−0.03 (0.01)	−0.06–0.00	−0.037	0.027
	Eat meals together	0.06 (0.02)	0.03−0.09	0.058	0.001

Model Summary—F 6.48; df 2; *p* < 0.01; R^2^ 0.003; ^1^ Confidence Interval (CI).

## Data Availability

Data that support the findings from this study are available. However, restrictions apply to the availability of data which were used under license for the current study and are thus, not publicly available. Data may be made available based on reasonable request and with permission of the Israel HBSC Program.
